# Deintensification of Treatment With Sulfonylurea and Insulin After Severe Hypoglycemia Among Older Adults With Diabetes

**DOI:** 10.1001/jamanetworkopen.2021.32215

**Published:** 2021-11-02

**Authors:** Anastasia-Stefania Alexopoulos, Anna R. Kahkoska, Virginia Pate, Marie C. Bradley, Joshua Niznik, Carolyn Thorpe, Til Stürmer, John Buse

**Affiliations:** 1 Department of Medicine, Division of Endocrinology, Duke University, Durham, North Carolina; 2 Durham Veterans Affairs Center of Innovation to Accelerate Discovery and Practice Transformation, Durham, North Carolina; 3 Department of Nutrition, University of North Carolina at Chapel Hill, Chapel Hill; 4 Department of Epidemiology, Gillings School of Global Public Health, University of North Carolina at Chapel Hill, Chapel Hill; 5 Division of Epidemiology, Office of Surveillance and Epidemiology, Center for Drug Evaluation and Research, Food and Drug Administration, Silver Spring, Maryland; 6 Department of Medicine, Division of Geriatrics and Center for Aging and Health, University of North Carolina at Chapel Hill, Chapel Hill; 7 Division of Pharmaceutical Outcomes and Policy, Eshelman School of Pharmacy, University of North Carolina at Chapel Hill, Chapel Hill; 8 Center of Health Equity Research and Promotion, Veterans Affairs Pittsburgh Healthcare System, Pittsburgh, Pennsylvania; 9 Department of Medicine, Division of Endocrinology, School of Medicine, University of North Carolina at Chapel Hill, Chapel Hill

## Abstract

**Importance:**

Practice guidelines recommend deintensification of hypoglycemic agents among older adults with diabetes who are at high risk of hypoglycemia, yet real-world treatment deintensification practices are not well characterized.

**Objective:**

To examine the incidence of sulfonylurea and insulin deintensification after a hypoglycemia-associated emergency department (ED) visit or hospitalization among older adults with diabetes and to identify factors associated with deintensification of treatment.

**Design, Setting, and Participants:**

This retrospective cohort study included a random sample of 20% of nationwide fee-for-service US Medicare beneficiaries aged 65 years and older with concurrent Medicare parts A, B, and D coverage between January 1, 2007, and December 31, 2017. Individuals with diabetes who had at least 1 hypoglycemia-associated ED visit or hospitalization were included. Data were analyzed from August 1, 2020, to August 1, 2021.

**Exposures:**

Baseline medication for the treatment of diabetes (sulfonylurea, insulin, or both).

**Main Outcomes and Measures:**

Incidence of treatment deintensification (yes or no) in the 100 days after a severe hypoglycemic episode requiring an ED visit or hospitalization, with treatment deintensification defined as (1) a decrease in sulfonylurea dose, (2) a change from long-acting to short-acting sulfonylurea (glipizide), (3) discontinuation of sulfonylurea, or (4) discontinuation of insulin based on pharmacy dispensing claims.

**Results:**

Among 76 278 distinct Medicare beneficiaries who had a hypoglycemia-associated ED visit or hospitalization, the mean (SD) age was 76.6 (7.6) years. Of 106 293 total hypoglycemic episodes requiring hospital attention, 69 084 (65.0%) occurred among women, 26 056 (24.5%) among Black individuals; 4761 (4.5%) among Hispanic individuals; 69 704 (65.6%) among White individuals; and 5772 (5.4%) among individuals of other races and ethnicities (comprising Asian, North American Native, unknown race or ethnicity, and unspecified race or ethnicity). A total of 32 074 episodes (30.2%) occurred among those receiving sulfonylurea only, 60 350 (56.8%) occurred among those receiving insulin only, and 13 869 (13.0%) occurred among those receiving both sulfonylurea and insulin. Treatment deintensification rates were highest among individuals receiving both sulfonylurea and insulin therapies at the time of their hypoglycemic episode (6677 episodes [48.1%]), followed by individuals receiving sulfonylurea only (14 192 episodes [44.2%]) and insulin only (14 495 episodes [24.0%]). Treatment deintensification rates increased between 2007 and 2017 (sulfonylurea only: from 41.4% to 49.7%; *P* < .001 for trend; insulin only: from 21.3% to 25.9%; *P* < .001 for trend; sulfonylurea and insulin: from 45.9% to 49.6%; *P* = .005 for trend). Lower socioeconomic status (as indicated by the receipt of low-income subsidies) was associated with lower odds of deintensification, regardless of baseline hypoglycemic regimen (sulfonylurea only: adjusted odds ratio [AOR], 0.74 [95% CI, 0.70-0.78]; insulin only: AOR, 0.71 [95% CI, 0.68-0.75]; sulfonylurea and insulin: AOR, 0.72 [95% CI, 0.66-0.78]). A number of patient factors were associated with higher odds of treatment deintensification: higher frailty (eg, ≥40% probability of needing assistance with activities of daily living among those receiving sulfonylurea and insulin: AOR, 1.50; 95% CI, 1.32-1.71), chronic kidney disease (eg, sulfonylurea and insulin: AOR, 1.29; 95% CI, 1.19-1.40), a history of falls (eg, sulfonylurea and insulin: AOR, 1.20; 95% CI, 1.09-1.33), and depression (eg, sulfonylurea and insulin: AOR, 1.11; 95% CI, 1.02-1.20).

**Conclusions and Relevance:**

In this cohort study, deintensification of sulfonylurea and/or insulin therapy within 100 days after a hypoglycemia-associated ED visit or hospitalization occurred in fewer than 50% of older adults with diabetes; however, these deintensification rates may be increasing over time, and deintensification of insulin was likely underestimated because of challenges in capturing changes to insulin dosing using administrative claims data. These results suggest that greater efforts are needed to identify individuals at high risk of hypoglycemia to encourage appropriate treatment deintensification in accordance with current evidence.

## Key Points

**Question:**

What is the incidence of sulfonylurea or insulin treatment deintensification among older adults after an episode of severe hypoglycemia?

**Findings:**

In this cohort study of 76 278 older adults with diabetes who had a hypoglycemia-associated emergency department visit or hospitalization while receiving sulfonylurea and/or insulin therapy, deintensification of the hypoglycemic treatment regimen occurred in fewer than 50% of episodes within 100 days after the event.

**Meaning:**

This study’s findings suggest that a gap exists between evidence-based and current practices regarding deintensification of hypoglycemia-inducing medications among older adults with diabetes who experienced severe hypoglycemia requiring an emergency department visit or hospitalization.

## Teaser

This cohort study uses Medicare claims data to assess the incidence of sulfonylurea and/or insulin treatment deintensification after an episode of severe hypoglycemia requiring an emergency department visit or hospitalization among older adults with diabetes.

## Introduction

Older adults with diabetes are at higher risk of hypoglycemia compared with younger populations,^1^ and this higher risk can lead to substantial morbidity and mortality.^2^ Other established factors associated with hypoglycemia that may further compound this risk among older adults include frailty, multimorbidity, and longer duration of diabetes,^3^ all of which are common in this population. Notably, the development of hypoglycemia among older adults is often associated with excessively intensive diabetes treatment regimens,^4^ particularly in those receiving sulfonylurea, glinide, and insulin therapies.^4,5^ Care guidelines recommend individualized deintensification of hypoglycemia-inducing medications in older adults with a high risk of hypoglycemia.^1^ However, despite evidence of safety and efficacy,^6^ multiple clinical and epidemiologic studies^7,8,9,10^ have suggested that treatment deintensification remains uncommon in this population, even among those with substantial frailty and multimorbidity.

Older adults with a history of severe hypoglycemia have an especially high risk of similar future events.^5^ Therefore, hypoglycemia-associated emergency department (ED) visits or hospitalizations among older adults should prompt close evaluation of the diabetes treatment regimen, and deintensification is likely reasonable in most cases. Despite the fact that hospitalization rates for hypoglycemia among older adults now exceed those for hyperglycemia (up to 105 admissions per 100 000 person-years),^11^ data on patterns of hypoglycemia-associated ED visits or hospitalizations are limited, and evidence thus far suggests no substantial change in prescription fills for diabetes medications after such an event.^10^ Robust data on real-world deintensification practices, including insight into the specific patient factors associated with treatment deintensification, are currently lacking.

An important step toward improving guideline-concordant care and reducing hypoglycemia-associated morbidity and mortality among older adults is to characterize treatment deintensification practices, particularly among those with the highest risk of severe hypoglycemia. To this end, our objective was to examine deintensification of treatment with sulfonylurea and insulin, the most common hypoglycemia-inducing medications, after a hypoglycemia-associated ED visit or hospitalization among older adults with diabetes and to identify patient factors associated with treatment deintensification.

## Methods

This study was approved by the institutional review board of the University of North Carolina at Chapel Hill, with a waiver of informed consent because of the use of deidentified data. The study protocol was registered with the European Network of Centres for Pharmacoepidemiology and Pharmacovigilance (registration number EUPAS37902) before accessing outcome data. This study followed the Strengthening the Reporting of Observational Studies in Epidemiology (STROBE) reporting guideline for cohort studies.^12^

### Study Population

We analyzed medical claims and prescription drug records to obtain a random sample of 20% of nationwide fee-for-service Medicare beneficiaries. The Medicare fee-for-service database includes health insurance claims for patients with Medicare part A (inpatient services), part B (outpatient care), and part D (outpatient prescription drug) coverage. We included beneficiaries aged 65 years and older who had at least 1 hypoglycemia-associated ED visit or hospitalization between January 1, 2007, and December 31, 2017 (eFigure in the Supplement), defined using the Ginde et al^13^ algorithm, with specificity greater than 99%. Consistent with previous studies,^5,13^ we adapted this algorithm to include codes from the *International Classification of Diseases, Tenth Revision, Clinical Modification* (eTable 1 in the Supplement). We required that hypoglycemia be in the primary coding position (vs any position) to further increase the likelihood that hypoglycemia was the predominant reason for presentation to the hospital. A hypoglycemia-associated ED visit or hospitalization was the index event, and each occurrence was treated as a separate index event, even if it occurred multiple times for the same patient. If claims for ED and hospital visits were submitted on consecutive days, they were collapsed into a single encounter. Episodes were categorized by date using an ordinal variable to signify first episode vs recurrent episodes.

Participants entered the cohort on the date of the index hypoglycemia-associated event. We included individuals with at least 1 prescription for sulfonylurea, insulin, or both sulfonylurea and insulin within 6 months before the index event. We excluded individuals without at least 6 months of continuous enrollment in Medicare parts A, B, and D before the index event and patients without 100 total days of follow-up.

### Outcomes

The primary outcome was deintensification of treatment with sulfonylurea and/or insulin in the 100 days after a hypoglycemia-associated ED visit or hospitalization. We defined deintensification as either (1) no prescription filled for baseline sulfonylurea and/or insulin within 100 days after the index event, (2) a prescription filled for sulfonylurea within 100 days after the index event at a lower dose than that dispensed in the last prescription before the index event, or (3) a change from long-acting sulfonylurea to glipizide within 100 days after the index event (Table 1).

**Table 1.** Sulfonylurea and Insulin Deintensification Definitions

Baseline treatment regimen	Prescription event
Sulfonylurea only	No sulfonylurea prescription filled within 100 d after index event
	Prescription filled for sulfonylurea within 100 d after index event at lower dose vs dose dispensed in last prescription before event^a^
	Change from long-acting sulfonylurea to glipizide within 100 d after index event^a^^,^^b^
Insulin only	No prescription filled for ≥1 type of baseline insulin (long-acting, short-acting, or both) within 100 d after index event
Insulin and sulfonylurea	No prescription filled for ≥1 type of baseline insulin (long-acting, short-acting, or both) within 100 d after index event
	Dose of sulfonylurea decreased and no change to insulin within 100 d after index event
	No prescription filled for sulfonylurea and no change to insulin within 100 d after index event
	Change from long-acting sulfonylurea to glipizide and no change to insulin^b^

^a^ Considered as deintensification only if insulin was not added to the treatment regimen within the same period (100 days after index event).

^b^ Long-acting sulfonylurea includes glimepiride, glyburide, and chlorpropamide.

The baseline (preindex event) treatment regimen was obtained using pharmacy dispensing claims data in the 100 days before a hypoglycemia-associated ED visit or hospitalization. We chose a relatively narrow period (approximately 3 months after the index event) to define treatment deintensification because a severe episode of hypoglycemia in an older adult typically prompts rapid review of the drug regimen by prescribers. The selection of 100 days (vs 90 days) was made to accommodate a 10-day period for detection of treatment deintensification among individuals who may have filled a 90-day prescription immediately before the index event.

### Patient Factors

Patient factors associated with sulfonylurea and/or insulin deintensification were assessed in the 6 months before each index event. We selected the following demographic and clinical characteristics based on their potential relevance to treatment deintensification: age, sex, self-reported race and ethnicity (Black, Hispanic, White, and other [including Asian, North American Native, unknown race or ethnicity, and unspecified race or ethnicity]), receipt of low-income subsidies (which provides payment assistance for beneficiaries with lower income), frailty (defined as the estimated probability of needing assistance with activities of daily living using the Faurot frailty measure^14^), diabetes complications, medical comorbidities (ie, cerebrovascular disease, chronic kidney disease, history of falls, cognitive impairment, dementia, arthritis, bladder dysfunction, chronic obstructive pulmonary disease, depression, alcohol misuse, cancer, hyperlipidemia, ischemic heart disease, and heart failure), receipt of medications relevant to diabetes care and hypoglycemia,^15^ and number of outpatient office visits during the study period (as a surrogate for health care use and access).

### Statistical Analysis

The demographic and clinical characteristics of Medicare beneficiaries with at least 1 hypoglycemia-associated ED visit or hospitalization were summarized using descriptive statistics (frequencies with percentages and means with SDs). We first quantified the unadjusted 100-day incidence of sulfonylurea and/or insulin deintensification after a hypoglycemia-associated ED visit or hospitalization in the entire cohort and according to baseline receipt of hypoglycemic medications (categorized as sulfonylurea only, insulin only, and sulfonylurea and insulin). We summarized the incidence by year to examine temporal changes in treatment deintensification.

We used multivariable logistic regression models to quantify the association between individual characteristics and the odds of treatment deintensification vs no treatment deintensification after a hypoglycemia-associated ED visit or hospitalization. The final model included demographic and clinical characteristics, presence of diabetes complications and comorbidities, receipt of glucose-lowering medications, receipt of other medications, and health care use and access (measured by the number of office visits). Dementia, arthritis, bladder dysfunction, hyperlipidemia, and heart failure were correlated with the Faurot frailty index (*r* > 0.5)^14^ and removed from the final models, which had a maximum variance inflation factor of 1.5. To aid in the interpretation of insulin deintensification rates given the limitations in capturing changes in insulin dosing using claims data, we constructed a parallel analysis in an independent cohort using an index event (ie, cataract surgery) that was unlikely to impact diabetes treatment. The goal of this sensitivity analysis was to describe the background insulin deintensification rates among a sample of older adults with diabetes who were not defined by a high risk of hypoglycemia. We expected the rates of insulin deintensification in the cataract surgery cohort to be reflective of rates that may occur at random in the population and may be lower than those observed in the hypoglycemia cohort.

Data were analyzed using SAS software, version 9.4 (SAS Institute), from August 1, 2020, to August 1, 2021. The statistical significance threshold was 2-sided *P* = .05.

## Results

### Study Sample

The study cohort comprised 76 278 distinct Medicare beneficiaries (eFigure in the Supplement), with a mean (SD) age of 76.6 (7.6) years. Among 106 293 total hypoglycemic episodes requiring hospital attention, 69 084 (65.0%) occurred among women, 37 209 (35.0%) among men, 26 056 (24.5%) among Black individuals, 4761 (4.5%) among Hispanic individuals, 69 704 (65.6%) among White individuals, and 5772 (5.4%) among individuals of other races and ethnicities (including Asian, North American Native, unspecified race or ethnicity, and unknown race or ethnicity) (Table 2). A total of 32 074 episodes (30.2%) occurred among those receiving sulfonylurea only, 60 350 (56.8%) occurred among those receiving insulin only, and 13 869 (13.0%) occurred among those receiving both sulfonylurea and insulin. Compared with individuals receiving sulfonylurea only, a higher proportion of individuals receiving insulin only were frail (eg, ≥40% probability of needing assistance with activities of daily living: 7207 episodes [22.5%] vs 15 026 episodes [24.9%], respectively) and had diabetes complications (17 677 episodes [55.1%] vs 58 520 episodes [97.0%]), chronic kidney disease (15 840 episodes [49.4%] vs 34 724 episodes [57.5%]), dementia (7982 episodes [24.9%] vs 15 955 episodes [26.4%]), chronic obstructive pulmonary disease (9227 episodes [28.8%] vs 18 800 episodes [31.2%]), depression (6012 episodes [18.7%] vs 15 199 episodes [25.2%]), and ischemic heart disease (15 180 episodes [47.3%] vs 32 934 [54.6%]).

**Table 2.** Participant Characteristics Stratified by Baseline Treatment Regimen

Characteristic	Episodes, No. (%)			
	All (N = 106 293)^a^	Sulfonylurea only (n = 32 074)^a^	Insulin only (n = 60 350)^a^	Sulfonylurea and insulin (n = 13 869)^a^
Previous severe hypoglycemic events, mean (SD), No.	0.6 (1.5)	0.2 (0.7)	0.8 (1.8)	0.4 (1.0)
Age, mean (SD), y	76.6 (7.6)	77.8 (7.6)	75.9 (7.5)	76.7 (7.7)
Sex				
Male	37 209 (35.0)	11 040 (34.4)	21 217 (35.2)	4952 (35.7)
Female	69 084 (65.0)	21 034 (65.6)	39 133 (64.8)	8917 (64.3)
Race and ethnicity				
Black	26 056 (24.5)	7469 (23.3)	15 161 (25.1)	3426 (24.7)
Hispanic	4761 (4.5)	1627 (5.1)	2403 (4.0)	731 (5.3)
White	69 704 (65.6)	20 562 (64.1)	40 225 (66.7)	8917 (64.3)
Other^b^	5772 (5.4)	2416 (7.5)	2561 (4.2)	795 (5.7)
No low-income subsidy	43 314 (40.7)	14 012 (43.7)	24 443 (40.5)	4859 (35.0)
Probability of ADL dependency, %				
<5	19 366 (18.2)	6170 (19.2)	10 889 (18.0)	2307 (16.6)
5-9	22 847 (21.5)	6905 (21.5)	13 257 (22.0)	2685 (19.4)
10-19	20 349 (19.1)	6437 (20.1)	11 317 (18.8)	2595 (18.7)
20-39	17 565 (16.5)	5355 (16.7)	9861 (16.3)	2349 (16.9)
≥40	26 166 (24.6)	7207 (22.5)	15 026 (24.9)	3933 (28.4)
Diabetes complications				
Nephropathy	21 149 (19.9)	4732 (14.8)	13 711 (22.7)	2706 (19.5)
Neuropathy	35 783 (33.7)	7657 (23.9)	23 401 (38.8)	4725 (34.1)
Retinopathy	30 469 (28.7)	5288 (16.5)	21 408 (35.5)	3773 (27.2)
Comorbidities				
Cerebrovascular disease	34 454 (32.4)	10 132 (31.6)	19 621 (32.5)	4701 (33.9)
Chronic kidney disease	58 344 (54.9)	15 840 (49.4)	34 724 (57.5)	7780 (56.1)
Cognitive impairment	2630 (2.5)	624 (1.9)	1662 (2.8)	344 (2.5)
Dementia	28 073 (26.4)	7982 (24.9)	15 955 (26.4)	4136 (29.8)
Chronic obstructive pulmonary disease	32 573 (30.6)	9227 (28.8)	18 800 (31.2)	4546 (32.8)
Depression	24 711 (23.2)	6012 (18.7)	15 199 (25.2)	3500 (25.2)
Alcohol misuse	1929 (1.8)	585 (1.8)	1098 (1.8)	246 (1.8)
History of falls	16 499 (15.5)	4577 (14.3)	9846 (16.3)	2076 (15.0)
Cancer	28 567 (26.9)	9000 (28.1)	15 894 (26.3)	3673 (26.5)
Ischemic heart disease	55 576 (52.3)	15 180 (47.3)	32 934 (54.6)	7462 (53.8)
Heart failure	42 114 (39.6)	11 804 (36.8)	24 297 (40.3)	6013 (43.4)
Diabetes medications				
Metformin	32 889 (30.9)	15 794 (49.2)	11 603 (19.2)	5492 (39.6)
Thiazolidinedione	11 717 (11.0)	5938 (18.5)	3806 (6.3)	1973 (14.2)
DPP-4 inhibitor	9011 (8.5)	3678 (11.5)	3498 (5.8)	1835 (13.2)
GLP-1 receptor agonist	1407 (1.3)	291 (0.9)	854 (1.4)	262 (1.9)
SGLT-2 inhibitor	364 (0.3)	78 (0.2)	214 (0.4)	72 (0.5)
Other medications				
Aspirin	9937 (9.3)	1001 (3.1)	7519 (12.5)	1417 (10.2)
Statin	71 438 (67.2)	21 023 (65.5)	40 760 (67.5)	9655 (69.6)
β-blocker	67 894 (63.9)	18 671 (58.2)	39 990 (66.3)	9233 (66.6)
ACEI	51 816 (48.7)	16 520 (51.5)	28 117 (46.6)	7179 (51.8)
ARB	27 133 (25.5)	8242 (25.7)	15 367 (25.5)	3524 (25.4)
CCB	43 918 (41.3)	13 213 (41.2)	24 795 (41.1)	5910 (42.6)
Glucocorticoid	21 726 (20.4)	6263 (19.5)	12 501 (20.7)	2962 (21.4)

Abbreviations: ACEI, angiotensin-converting enzyme inhibitor; ADL, activities of daily living; ARB, angiotensin II receptor blocker; CCB, calcium channel blocker; DPP-4, dipeptidyl peptidase 4; GLP-1, glucagon-like peptide 1; SGLT-2, sodium/glucose cotransporter 2.

^a^ Number of unique beneficiaries.

^b^ Other self-identified races and ethnicities included Asian, North American Native, unknown race or ethnicity, and unspecified race or ethnicity.

A higher proportion of individuals with multiple vs single hypoglycemia-associated ED visits or hospitalizations self-identified as Black (5118 of 17 702 individuals [28.9%] vs 11 423 of 58 579 individuals [19.5%], respectively) and were receiving insulin-only treatment regimens (10 587 of 17 702 individuals [59.8%] vs 28 658 of 58 579 individuals [48.9%]) (eTable 2 in the Supplement). Among the total study cohort, 10 702 individuals (10.1%) received sulfonylurea and/or insulin prescriptions from an endocrinologist, and most of those individuals (8487 [79.3%]) were receiving insulin only (eTable 3 and eTable 4 in the Supplement). Within each baseline treatment regimen, deintensification rates were similar across prescriber types (eg, sulfonylurea only: 43.6% for endocrinologists, 44.3% for primary care physicians, and 43.9% for other specialists) (eTable 5 in the Supplement).

A total of 15 696 episodes were excluded from our cohort because the individuals died, representing an all-cause 100-day mortality rate of 11.5% after a hypoglycemia-associated ED visit or hospitalization (eFigure in the Supplement).

### Incidence of Deintensification

The unadjusted incidence of treatment deintensification after a hypoglycemia-associated ED visit or hospitalization was highest among individuals receiving both sulfonylurea and insulin (6677 episodes [48.1%]), followed by those receiving sulfonylurea only (14 192 episodes [44.2%]) (Table 3). The lowest incidence of deintensification was observed among older adults receiving insulin only (14 495 episodes [24.0%]). Deintensification rates increased between 2007 and 2017 (sulfonylurea only: from 41.4% to 49.7%; *P* < .001 for trend; insulin only: from 21.3% to 25.9%; *P* < .001 for trend; sulfonylurea and insulin: from 45.9% to 49.6%; *P* = .005 for trend) (Figure).

**Table 3.** Unadjusted Deintensification Rates Stratified by Baseline Treatment Regimen

Characteristic	No./total No. (%)		
	Sulfonylurea only	Insulin only	Sulfonylurea and insulin
Total episodes, No.	32 074	60 350	13 869
Deintensification of treatment	14 192/32 074 (44.2)^a^	14 495/60 350 (24.0)	6677/13 869 (48.1)
Hypoglycemic events			
1	11 728/26 764 (43.8)	9419/39 244 (24.0)	4858/10 270 (47.3)
≥2	2464/5310 (46.4)	5076/21 106 (24.1)	1819/3599 (50.5)
Age range, y			
65-75	5491/13 187 (41.6)	7500/31 105 (24.1)	3105/6640 (46.8)
>75	8701/18 887 (46.1)	6995/29 245 (23.9)	3572/7229 (49.4)
Sex			
Male	4937/11 040 (44.7)	5265/21 217 (24.8)	2372/4952 (47.9)
Female	9255/21 034 (44.0)	9230/39 133 (23.6)	4305/8917 (48.3)
Race and ethnicity			
Black	3079/7469 (41.2)	3741/15 161 (24.7)	1682/3426 (49.1)
Hispanic	645/1627 (39.6)	552/2403 (23.0)	382/731 (52.3)
White	9422/20 562 (45.8)	9585/40 225 (23.8)	4242/8917 (47.6)
Other^b^	1046/2416 (43.3)	617/2561 (24.1)	371/795 (46.7)

^a^ A total of 12 896 individuals (90.9%) discontinued receipt of sulfonylurea, 750 individuals (5.3%) decreased the dose of sulfonylurea, and 546 individuals (3.8%) changed from long-acting sulfonylurea to glipizide.

^b^ Other self-identified races and ethnicities included Asian, North American Native, unspecified race or ethnicity, and unknown race or ethnicity.

**Figure.**
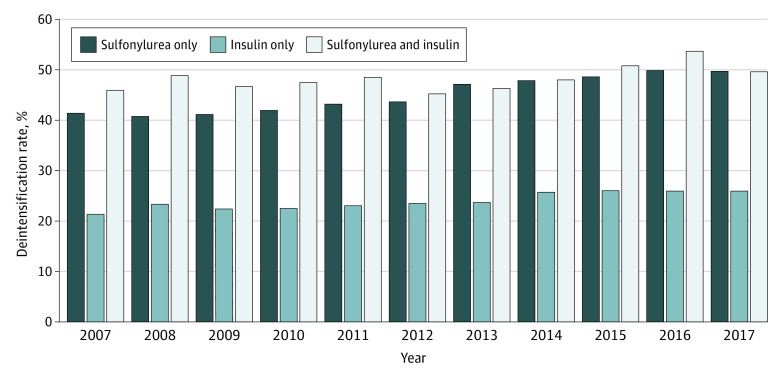
Temporal Changes in Deintensification of Sulfonylurea and Insulin Therapies After Severe Hypoglycemia

### Individual Characteristics Associated With Deintensification

Compared with patients aged 65 to 75 years, those older than 75 years had slightly lower odds of deintensification if they were receiving insulin only (adjusted odds ratio [AOR], 0.94; 95% CI, 0.90-0.98) (Table 4). Older adults of Black race had lower odds of deintensification if they were receiving sulfonylurea only (AOR, 0.88; 95% CI, 0.83-0.94). In contrast, Black and Hispanic individuals had higher odds of deintensification if they were receiving insulin only (Black: AOR, 1.15 [95% CI, 1.10-1.21]; Hispanic: AOR, 1.12 [95% CI, 1.01-1.24]) or both sulfonylurea and insulin (Black: AOR, 1.15 [95% CI, 1.05-1.25]; Hispanic: AOR, 1.45 [95% CI, 1.24-1.71]). Individuals who were receiving low-income subsidies had lower odds of treatment deintensification (sulfonylurea only: AOR, 0.74 [95% CI, 0.70-0.78]; insulin only: AOR, 0.71 [95% CI, 0.68-0.75]; sulfonylurea and insulin: AOR, 0.72 [95% CI, 0.66-0.78]) compared with those who were not receiving low-income subsidies.

**Table 4.** Unadjusted and Adjusted Odds Ratios of Deintensification After Severe Hypoglycemia Stratified by Baseline Treatment Regimen

Variable	Sulfonylurea only		Insulin only		Sulfonylurea and insulin	
	OR (95% CI)	AOR (95% CI)	OR (95% CI)	AOR (95% CI)	OR (95% CI)	AOR (95% CI)
≥2 Hypoglycemic events	1.11 (1.05-1.18)	1.10 (1.03-1.17)	1.00 (0.96-1.04)	0.98 (0.94-1.02)	1.14 (1.06-1.23)	1.09 (1.01-1.18)
Age >75 y	1.20 (1.14-1.25)	1.08 (1.02-1.13)	0.99 (0.95-1.03)	0.94 (0.90-0.98)	1.11 (1.04-1.19)	1.03 (0.95-1.11)
Female sex	0.97 (0.93-1.02)	1.01 (0.96-1.06)	0.94 (0.90-0.97)	0.99 (0.95-1.03)	1.02 (0.95-1.09)	1.02 (0.94-1.10)
Race and ethnicity						
Black	0.83 (0.79-0.87)	0.88 (0.83-0.94)	1.05 (1.00-1.09)	1.15 (1.10-1.21)	1.06 (0.98-1.15)	1.15 (1.05-1.25)
Hispanic	0.78 (0.70-0.86)	0.93 (0.83-1.04)	0.95 (0.86-1.05)	1.12 (1.01-1.24)	1.21 (1.04-1.40)	1.45 (1.24-1.71)
White	1 [Reference]	1 [Reference]	1 [Reference]	1 [Reference]	1 [Reference]	1 [Reference]
Other^a^	0.90 (0.83-0.98)	1.01 (0.92-1.10)	1.01 (0.92-1.11)	1.08 (0.98-1.19)	0.96 (0.83-1.12)	1.02 (0.88-1.19)
Receiving low-income subsidy	0.73 (0.70-0.77)	0.74 (0.70-0.78)	0.79 (0.76-0.82)	0.71 (0.68-0.75)	0.83 (0.77-0.89)	0.72 (0.66-0.78)
Probability of ADL dependency, %						
<5	1 [Reference]	1 [Reference]	1 [Reference]	1 [Reference]	1 [Reference]	1 [Reference]
5-9	1.11 (1.04-1.19)	1.07 (1.00-1.15)	1.07 (1.01-1.14)	1.06 (0.99-1.13)	1.28 (1.14-1.43)	1.26 (1.13-1.42)
10-19	1.27 (1.18-1.36)	1.20 (1.11-1.30)	1.06 (1.00-1.13)	1.06 (0.99-1.13)	1.24 (1.11-1.39)	1.22 (1.08-1.38)
20-39	1.36 (1.26-1.46)	1.27 (1.17-1.38)	1.16 (1.09-1.24)	1.15 (1.07-1.23)	1.34 (1.19-1.50)	1.31 (1.15-1.49)
≥40	1.52 (1.42-1.63)	1.38 (1.27-1.50)	1.37 (1.29-1.45)	1.31 (1.22-1.41)	1.59 (1.43-1.76)	1.50 (1.32-1.71)
Diabetes complications						
Nephropathy	1.16 (1.09-1.24)	1.00 (0.93-1.07)	1.32 (1.26-1.38)	1.13 (1.07-1.18)	1.23 (1.13-1.33)	1.09 (0.99-1.20)
Neuropathy	1.04 (0.98-1.09)	0.96 (0.91-1.01)	1.13 (1.08-1.17)	1.01 (0.97-1.05)	1.01 (0.94-1.08)	0.90 (0.84-0.97)
Retinopathy	0.99 (0.93-1.05)	0.97 (0.91-1.03)	1.03 (0.99-1.08)	0.97 (0.93-1.01)	0.99 (0.92-1.07)	0.96 (0.89-1.04)
Comorbidities						
Chronic kidney disease	1.38 (1.32-1.44)	1.34 (1.28-1.42)	1.38 (1.33-1.43)	1.26 (1.21-1.32)	1.35 (1.26-1.44)	1.29 (1.19-1.40)
Cerebrovascular disease	1.21 (1.15-1.26)	1.09 (1.03-1.14)	1.23 (1.18-1.28)	1.11 (1.06-1.15)	1.16 (1.08-1.25)	1.03 (0.95-1.11)
History of falls	1.26 (1.18-1.34)	1.11 (1.04-1.19)	1.18 (1.12-1.24)	1.08 (1.03-1.14)	1.30 (1.18-1.43)	1.20 (1.09-1.33)
Cognitive impairment	1.41 (1.20-1.65)	1.17 (0.99-1.37)	1.19 (1.07-1.33)	0.99 (0.88-1.10)	1.23 (0.99-1.53)	1.07 (0.86-1.33)
Chronic obstructive pulmonary disease	1.08 (1.03-1.13)	0.97 (0.92-1.02)	1.14 (1.10-1.19)	1.04 (1.00-1.09)	1.12 (1.04-1.20)	1.02 (0.94-1.10)
Depression	1.21 (1.14-1.28)	1.10 (1.03-1.17)	1.14 (1.10-1.19)	1.06 (1.01-1.11)	1.21 (1.12-1.31)	1.11 (1.02-1.20)
Alcohol misuse	1.24 (1.06-1.46)	1.23 (1.04-1.45)	1.22 (1.06-1.39)	1.12 (0.98-1.29)	1.48 (1.15-1.91)	1.39 (1.07-1.80)
Cancer	1.19 (1.14-1.25)	1.10 (1.05-1.16)	1.17 (1.12-1.22)	1.09 (1.04-1.13)	1.07 (1.00-1.16)	1.01 (0.93-1.09)
Ischemic heart disease	1.16 (1.11-1.22)	1.06 (1.01-1.11)	1.21 (1.16-1.25)	1.10 (1.05-1.15)	1.15 (1.07-1.23)	1.07 (0.99-1.15)
Diabetes medications						
Metformin	0.90 (0.86-0.94)	1.03 (0.98-1.08)	0.98 (0.94-1.03)	1.10 (1.05-1.16)	1.07 (1.00-1.15)	1.21 (1.13-1.30)
Thiazolidinedione	0.84 (0.79-0.88)	0.91 (0.86-0.97)	0.97 (0.90-1.05)	1.06 (0.98-1.15)	1.03 (0.94-1.13)	1.10 (1.00-1.21)
DPP-4 inhibitor	0.89 (0.83-0.96)	0.90 (0.83-0.96)	1.02 (0.94-1.10)	1.01 (0.93-1.09)	1.03 (0.94-1.14)	1.03 (0.93-1.14)
GLP-1 receptor agonist	0.83 (0.66-1.06)	0.86 (0.67-1.09)	0.99 (0.85-1.16)	1.02 (0.86-1.19)	0.94 (0.73-1.20)	1.00 (0.78-1.28)
SGLT-2 inhibitor	0.92 (0.59-1.45)	1.00 (0.63-1.58)	0.87 (0.62-1.20)	0.85 (0.61-1.19)	0.96 (0.61-1.53)	0.94 (0.59-1.51)
Other medications						
Aspirin	1.02 (0.90-1.15)	0.92 (0.81-1.05)	1.02 (0.97-1.08)	1.02 (0.96-1.08)	1.02 (0.91-1.13)	1.01 (0.91-1.13)
Statin	0.91 (0.87-0.95)	0.93 (0.89-0.98)	0.92 (0.88-0.96)	0.91 (0.87-0.95)	0.92 (0.86-0.99)	0.94 (0.87-1.01)
β-blocker	1.02 (0.97-1.06)	0.95 (0.91-1.00)	1.05 (1.01-1.09)	0.97 (0.93-1.01)	0.99 (0.92-1.06)	0.93 (0.86-1.01)
ACEI	0.92 (0.88-0.96)	0.93 (0.88-0.98)	0.92 (0.89-0.96)	0.92 (0.88-0.96)	0.98 (0.92-1.05)	0.93 (0.86-1.00)
ARB	0.98 (0.93-1.03)	0.97 (0.91-1.02)	0.92 (0.88-0.96)	0.89 (0.85-0.93)	0.87 (0.81-0.94)	0.82 (0.75-0.89)
CCB	1.02 (0.98-1.07)	1.01 (0.97-1.06)	1.03 (0.99-1.07)	0.99 (0.95-1.03)	1.09 (1.02-1.16)	1.06 (0.98-1.13)
Glucocorticoid	1.05 (1.00-1.11)	1.02 (0.96-1.08)	1.08 (1.03-1.13)	1.02 (0.97-1.07)	1.11 (1.02-1.20)	1.07 (0.99-1.17)
Office visits						
<1	1 [Reference]	1 [Reference]	1 [Reference]	1 [Reference]	1 [Reference]	1 [Reference]
1-2	0.97 (0.88-1.07)	0.98 (0.89-1.09)	1.03 (0.95-1.12)	1.04 (0.96-1.13)	0.97 (0.85-1.11)	1.00 (0.88-1.15)
3-5	0.97 (0.88-1.07)	0.96 (0.87-1.05)	1.06 (0.98-1.14)	1.05 (0.98-1.14)	0.91 (0.80-1.03)	0.93 (0.82-1.06)
6-10	1.04 (0.95-1.14)	0.97 (0.88-1.07)	1.13 (1.05-1.22)	1.07 (0.99-1.15)	1.06 (0.94-1.20)	1.04 (0.91-1.18)
>11	1.10 (0.99-1.22)	0.96 (0.86-1.07)	1.24 (1.15-1.35)	1.08 (0.99-1.18)	1.00 (0.88-1.15)	0.94 (0.81-1.09)

Abbreviations: ACEI, angiotensin-converting enzyme inhibitor; ADL, activities of daily living; AOR, adjusted odds ratio; ARB, angiotensin II receptor blocker; CCB, calcium channel blocker; DPP-4, dipeptidyl peptidase 4; GLP-1, glucagon-like peptide 1; OR, odds ratio; SGLT-2, sodium/glucose cotransporter 2.

^a^ Other self-identified races and ethnicities included Asian, North American Native, unspecified race or ethnicity, and unknown race or ethnicity.

Certain clinical factors were also associated with treatment deintensification. Compared with the first episode of severe hypoglycemia, recurrent episodes (≥2) were associated with only modestly higher odds of deintensification, and those higher odds were observed only among individuals receiving sulfonylurea only (AOR, 1.10; 95% CI, 1.03-1.17) or sulfonylurea in combination with insulin (AOR, 1.09; 95% CI, 1.01-1.18). Higher odds of deintensification across all baseline treatment regimens were found among those with greater frailty (eg, ≥40% probability of needing assistance with activities of daily living among those receiving sulfonylurea only: AOR, 1.38 [95% CI, 1.27-1.50]; insulin only: AOR, 1.31 [95% CI, 1.22-1.41]; and sulfonylurea and insulin: AOR, 1.50 [95% CI, 1.32-1.71]). Higher odds of treatment deintensification were also observed among patients with a history of chronic kidney disease (sulfonylurea only: AOR, 1.34 [95% CI, 1.28-1.42]; insulin only: AOR, 1.26 [95% CI, 1.21-1.32]; sulfonylurea and insulin: AOR, 1.29 [95% CI, 1.19-1.40]), and, to a lesser extent, among older adults with a history of falls (sulfonylurea only: AOR, 1.11 [95% CI, 1.04-1.19]; insulin only: AOR, 1.08 [95% CI, 1.03-1.14]; sulfonylurea and insulin: AOR, 1.20 [95% CI, 1.09-1.33]) or depression (sulfonylurea only: AOR, 1.10 [95% CI, 1.03-1.17]; insulin only: AOR, 1.06 [95% CI, 1.01-1.11]; sulfonylurea and insulin: AOR, 1.11 [95% CI 1.02-1.20]). Similar results were noted for patients with a history of cerebrovascular disease [eg, sulfonylurea only: AOR, 1.09 [95% CI, 1.03-1.14]; insulin only: AOR, 1.11 [95% CI, 1.06-1.15]), alcohol misuse (eg, sulfonylurea only: AOR, 1.23 [95% CI, 1.04-1.45]; sulfonylurea and insulin: AOR, 1.39 [95% CI, 1.07-1.80]), cancer (eg, sulfonylurea only: AOR, 1.10 [95% CI, 1.05-1.16]; insulin only: AOR, 1.09 [95% CI, 1.04-1.13]), and ischemic heart disease (eg, sulfonylurea only: AOR, 1.06 [95% CI, 1.01-1.11]; insulin only: AOR, 1.10 [95% CI, 1.05-1.15]); however, statistical significance was not achieved consistently across baseline regimens.

With regard to medications, concomitant receipt of angiotensin-converting enzyme inhibitors (sulfonylurea only: AOR, 0.93 [95% CI, 0.88-0.98]; insulin only: AOR, 0.92 [95% CI, 0.88-0.96]; sulfonylurea and insulin: AOR, 0.93 [95% CI, 0.86-1.00]), angiotensin II receptor blockers (sulfonylurea only: AOR, 0.97 [95% CI, 0.91-1.02]; insulin only: AOR, 0.89 [95% CI, 0.85-0.93]; sulfonylurea and insulin: AOR, 0.82 [95% CI, 0.75-0.89]), and statins (sulfonylurea only: AOR, 0.93 [95% CI, 0.89-0.98]; insulin only: AOR, 0.91 [95% CI, 0.87-0.95]; sulfonylurea and insulin: AOR, 0.94 [95% CI, 0.87-1.01]) were associated with lower odds of treatment deintensification after severe hypoglycemia. Baseline metformin receipt was associated with higher odds of deintensification of insulin-containing treatment regimens (insulin only: AOR, 1.10 [95% CI, 1.05-1.16]; sulfonylurea and insulin: AOR, 1.21 [95% CI, 1.13-1.30]).

Sensitivity analyses conducted using outpatient cataract surgery as an index event (eTable 6 in the Supplement) revealed lower deintensification rates overall (13.1% for sulfonylurea only, 17.5% for insulin only, and 28.2% for sulfonylurea and insulin) compared with the hypoglycemia cohort (44.2% for sulfonylurea only, 24.0% for insulin only, and 48.1% for sulfonylurea and insulin).

## Discussion

In this cohort study of an older adult population with diabetes, we found sulfonylurea and/or insulin deintensification rates of less than 50% after a hypoglycemia-associated ED visit or hospitalization, although insulin deintensification rates were likely underestimated because of the inability to capture changes in insulin dosing using claims data. Although there was a pattern of increasing sulfonylurea and/or insulin deintensification over a 10-year period, low overall rates of deintensification suggest that real-world practice may lag behind evidence that positions severe hypoglycemia as a major health and safety concern among older adults.

We found that 11.5% of the Medicare cohort died within the 100 days after hospital presentation for hypoglycemia (eFigure in the Supplement). This finding is consistent with existing knowledge that suggests an association between severe hypoglycemia and all-cause mortality,^16,17,18^ and it underscores the importance of hypoglycemia avoidance in this population. Our deintensification definition did not allow us to discern whether patients who died experienced treatment deintensification before death; therefore, we had to base our analysis on survival.

Older adults with severe hypoglycemia are at a high risk of repeated events^5^; thus, in most cases, consideration of deintensification of hypoglycemia-inducing agents is warranted after an ED visit or hospitalization for hypoglycemia. Yet, rates of sulfonylurea and/or insulin deintensification were lower than 50% in our study, and the odds of deintensification were not appreciably higher among individuals with repeated episodes (vs 1 episode) of severe hypoglycemia requiring hospital attention or among individuals older than 75 years. Our findings were similar to those of previous studies^7,8,9,10,19^ that reported low rates of treatment deintensification among older adults, including adults with frailty, multimorbidity, and low life expectancy. These data suggest a need for enhanced recognition of individuals at high risk for recurrent hypoglycemic episodes as well as efforts to combat clinical inertia associated with treatment deintensification in this setting.

We found that treatment deintensification practices varied by race and ethnicity. Compared with non-Hispanic White individuals, Black individuals had lower odds of deintensification for sulfonylurea only. However, Hispanic and Black individuals had higher odds of deintensification of insulin-containing treatment regimens. At this time, data on racial and ethnic disparities in deintensification of hypoglycemia-inducing agents are limited. One study by Maciejewski et al^20^ categorized older adults with diabetes as potentially overtreated or undertreated (by glycated hemoglobin A_1c_ and medication criteria), and they did not find race or ethnicity to be associated with the odds of treatment deintensification in the overtreated group. However, similar to other studies,^20,21,22^ Maciejewski et al^20^ found Black and Hispanic patients to be at a significantly higher risk of undertreatment for diabetes. Our observational study design was not ideal to draw inferences about the appropriateness of deintensification on a case-by-case basis. Further work is necessary to understand these associations and to identify and correct health disparities that may place specific racial and ethnic subgroups at a higher risk of future hypoglycemic events.

We also found chronic kidney disease, a history of falls, and depression to be associated with significantly more frequent deintensification, regardless of baseline treatment regimen. Similar findings were observed for cerebrovascular disease and ischemic heart disease. All of these comorbidities are associated with a markedly high risk of severe hypoglycemia,^5^ suggesting that health care professionals may identify and act upon high-risk clinical phenotypes, including those that would be most affected by the sequelae of hypoglycemia. Consistent with recommended practices,^1^ we further observed a consistent pattern of more frequent sulfonylurea and/or insulin deintensification among older adults with higher markers of frailty. This finding is in contrast to those of a study in which insulin discontinuation among adults aged 75 to 79 years was most common in healthier individuals vs those with multimorbidity.^9^ However, treatment deintensification increased only modestly with increasing frailty, so there likely remain many missed opportunities for deintensification when clinically indicated.

Notably, a lower likelihood of deintensification was found among patients receiving angiotensin-converting enzyme inhibitors and angiotensin II receptor blockers as well as statins, which suggests that patients with diabetes complications that were being managed more aggressively were less likely to experience treatment deintensification.

We observed a lower incidence of, and variability in, insulin deintensification compared with sulfonylurea deintensification. This difference may be due to decreased sensitivity in our insulin deintensification classification because of the methodologic challenges associated with capturing changes in insulin dosing in claims data. In the present study, insulin deintensification was defined as discontinuation of at least 1 type of insulin received at baseline. Although this is a standard approach used by other researchers,^6,8,19,20^ it does not capture treatment deintensification in a scenario in which a prescriber advises lowering the insulin dose but does not discontinue insulin altogether. A sensitivity analysis of unadjusted insulin deintensification rates in an independent cohort that was not expected to have undergone medication changes (ie, patients after outpatient cataract surgery) found insulin deintensification rates to be approximately 7% lower. Together, these data reveal opportunities for future studies to develop and refine methods to more accurately capture insulin deintensification.

### Strengths and Limitations

This study has several strengths. We used a large, nationally representative population to identify patterns in sulfonylurea and insulin deintensification and examine changes in deintensification practices over a 10-year period. Our definition of sulfonylurea deintensification included changing from a long-acting to a short-acting sulfonylurea, which allowed a more inclusive assessment of prescriber practices aimed at lowering future hypoglycemia risk. We also examined a wide range of patient factors that may be associated with deintensification, and these data can be used to inform interventions to promote evidence-based care as it pertains to minimizing hypoglycemia in older adults.

This study also has limitations. This cohort did not include patients with severe hypoglycemia who did not require hospital attention; thus, the findings may not be generalizable to individuals who experience such episodes but are treated at home. We may not have detected deintensification that occurred before death within 100 days. In addition, although individuals receiving sulfonylurea therapy are likely to have type 2 diabetes, those who receive insulin only may include individuals with type 1 diabetes for whom insulin deintensification or discontinuation may be inappropriate. However, individuals with type 1 diabetes likely make up only a small sample of the Medicare population with diabetes. In addition, we were not able to assess hemoglobin A_1c_, although an association between hemoglobin A_1c_ and hypoglycemia-associated ED visits or hospitalizations has not been found.^10^ Furthermore, even among those with high hemoglobin A_1c_ levels, severe hypoglycemia is a potentially life-threatening complication that requires a change in therapy in most cases, particularly among older adults for whom the short-term consequences of severe hypoglycemia substantially exceed those of hyperglycemia. Because we used pharmacy dispensing claims to define treatment deintensification, we also do not know whether deintensification was the result of a prescription change by the prescriber or the possibility that the patient did not fill the prescription. In addition, it is not possible to draw conclusions regarding the appropriateness of deintensification (or lack thereof) for the individual cases reflected in these data.

## Conclusions

This cohort study found that, although the rates of treatment deintensification slowly improved between 2007 and 2017, deintensification of sulfonylurea and/or insulin therapy within 100 days after a hypoglycemia-associated ED visit or hospitalization occurred for fewer than 50% of older adults with diabetes. These findings suggest that greater efforts are needed to identify individuals at high risk of recurrent hypoglycemia and to encourage appropriate and equitable treatment deintensification practices for patients hospitalized with hypoglycemia to reduce hypoglycemia-associated morbidity and mortality.

## Notes

Supplement. **eTable 1.** *ICD-9* and *ICD-10* Codes Used for Defining Hypoglycemia-Associated ED Visits or Hospitalizations **eTable 2.** Characteristics of Individuals With 1 vs Multiple Episodes of Hypoglycemia-Associated ED Visits or Hospitalizations **eTable 3.** Specialty of Prescribers of Sulfonylurea and/or Insulin Therapy **eTable 4.** Baseline Regimen by Prescriber Specialty **eTable 5.** Deintensification of Baseline Regimen by Prescriber Specialty **eTable 6.** Sensitivity Analysis of Outpatient Cataract Surgery Cohort **eFigure.** Flow Diagram of Study Population

## References

[1] American Diabetes Association. 12. Older adults: *Standards of Medical Care in Diabetes—2021.* Diabetes Care. 2021;44(suppl 1):S168-S179. doi:10.2337/dc21-S012 33298423

[2] International Hypoglycaemia Study Group. Minimizing hypoglycemia in diabetes. Diabetes Care. 2015;38(8):1583-1591. doi:10.2337/dc15-0279 26207052

[3] Silbert R, Salcido-Montenegro A, Rodriguez-Gutierrez R, Katabi A, McCoy RG. Hypoglycemia among patients with type 2 diabetes: epidemiology, risk factors, and prevention strategies. Curr Diabetes Rep. 2018;18(8):53. doi:10.1007/s11892-018-1018-0 29931579 PMC6117835

[4] Mahoney GK, Henk HJ, McCoy RG. Severe hypoglycemia attributable to intensive glucose-lowering therapy among US adults with diabetes: population-based modeling study, 2011-2014. Mayo Clin Proc. 2019;94(9):1731-1742. doi:10.1016/j.mayocp.2019.02.028 31422897 PMC6857710

[5] McCoy RG, Lipska KJ, Van Houten HK, Shah ND. Association of cumulative multimorbidity, glycemic control, and medication use with hypoglycemia-related emergency department visits and hospitalizations among adults with diabetes. JAMA Netw Open. 2020;3(1):e1919099. doi:10.1001/jamanetworkopen.2019.19099 31922562 PMC6991264

[6] Seidu S, Kunutsor SK, Topsever P, Hambling CE, Cos FX, Khunti K. Deintensification in older patients with type 2 diabetes: a systematic review of approaches, rates and outcomes. Diabetes Obes Metab. 2019;21(7):1668-1679. doi:10.1111/dom.13724 30938038

[7] Lipska KJ, Ross JS, Miao Y, Shah ND, Lee SJ, Steinman MA. Potential overtreatment of diabetes mellitus in older adults with tight glycemic control. JAMA Intern Med. 2015;175(3):356-362. doi:10.1001/jamainternmed.2014.7345 25581565 PMC4426991

[8] Sussman JB, Kerr EA, Saini SD, . Rates of deintensification of blood pressure and glycemic medication treatment based on levels of control and life expectancy in older patients with diabetes mellitus. JAMA Intern Med. 2015;175(12):1942-1949. doi:10.1001/jamainternmed.2015.5110 26502220 PMC9617259

[9] Weiner JZ, Gopalan A, Mishra P, . Use and discontinuation of insulin treatment among adults aged 75 to 79 years with type 2 diabetes. JAMA Intern Med. 2019;179(12):1633-1641. doi:10.1001/jamainternmed.2019.3759 31545376 PMC6763990

[10] Vijayakumar P, Liu S, McCoy RG, Karter AJ, Lipska KJ. Changes in management of type 2 diabetes before and after severe hypoglycemia. Diabetes Care. 2020;43(11):e188-e189. doi:10.2337/dc20-0458 32943439 PMC7576416

[11] Lipska KJ, Ross JS, Wang Y, . National trends in US hospital admissions for hyperglycemia and hypoglycemia among Medicare beneficiaries, 1999 to 2011. JAMA Intern Med. 2014;174(7):1116-1124. doi:10.1001/jamainternmed.2014.1824 24838229 PMC4152370

[12] von Elm E, Altman DG, Egger M, Pocock SJ, Gotzsche PC, Vandenbroucke JP; STROBE Initiative. The Strengthening the Reporting of Observational Studies in Epidemiology (STROBE) statement: guidelines for reporting observational studies. Int J Surg. 2014;12(12):1495-1499. doi:10.1016/j.ijsu.2014.07.013 25046131

[13] Ginde AA, Blanc PG, Lieberman RM, Camargo CA Jr. Validation of *ICD-9-CM* coding algorithm for improved identification of hypoglycemia visits. BMC Endocr Disord. 2008;8:4. doi:10.1186/1472-6823-8-4 18380903 PMC2323001

[14] Faurot KR, Jonsson Funk M, Pate V, . Using claims data to predict dependency in activities of daily living as a proxy for frailty. Pharmacoepidemiol Drug Saf. 2015;24(1):59-66. doi:10.1002/pds.3719 25335470 PMC4293227

[15] Vue MH, Setter SM. Drug-induced glucose alterations, part 1: drug-induced hypoglycemia. Diabetes Spectr. 2011;24(3):171-177. doi:10.2337/diaspect.24.3.171

[16] McCoy RG, Van Houten HK, Ziegenfuss JY, Shah ND, Wermers RA, Smith SA. Increased mortality of patients with diabetes reporting severe hypoglycemia. Diabetes Care. 2012;35(9):1897-1901. doi:10.2337/dc11-2054 22699297 PMC3425008

[17] Hanefeld M, Frier BM, Pistrosch F. Hypoglycemia and cardiovascular risk: is there a major link? Diabetes Care. 2016;39(suppl 2):S205-S209. doi:10.2337/dcS15-3014 27440834

[18] Bonds DE, Miller ME, Bergenstal RM, . The association between symptomatic, severe hypoglycaemia and mortality in type 2 diabetes: retrospective epidemiological analysis of the ACCORD study. BMJ. 2010;340:b4909. doi:10.1136/bmj.b4909 20061358 PMC2803744

[19] Niznik JD, Hunnicutt JN, Zhao X, . Deintensification of diabetes medications among veterans at the end of life in VA nursing homes. J Am Geriatr Soc. 2020;68(4):736-745. doi:10.1111/jgs.16360 32065387 PMC7456123

[20] Maciejewski ML, Mi X, Sussman J, . Overtreatment and deintensification of diabetic therapy among Medicare beneficiaries. J Gen Intern Med. 2018;33(1):34-41. doi:10.1007/s11606-017-4167-y 28905179 PMC5756160

[21] Ayanian JZ, Landon BE, Newhouse JP, Zaslavsky AM. Racial and ethnic disparities among enrollees in Medicare Advantage plans. N Engl J Med. 2014;371(24):2288-2297. doi:10.1056/NEJMsa1407273 25494268 PMC4381536

[22] Bonds DE, Zaccaro DJ, Karter AJ, Selby JV, Saad M, Goff DC Jr. Ethnic and racial differences in diabetes care: the Insulin Resistance Atherosclerosis Study. Diabetes Care. 2003;26(4):1040-1046. doi:10.2337/diacare.26.4.1040 12663570

