# Supplementary material for: Deintensification of Treatment With Sulfonylurea and Insulin After Severe Hypoglycemia Among Older Adults With Diabetes
Source: JAMA Netw Open. 2021 Nov 2;4(11):e2132215. doi: 10.1001/jamanetworkopen.2021.32215 (PMC8564578; doi:10.1001/jamanetworkopen.2021.32215)
Supplement: Supplement. — eTable 1. ICD-9 and ICD-10 Codes Used for Defining Hypoglycemia-Associated ED Visits or Hospitalizations eTable 2. Characteristics of Individuals With 1 vs Multiple Episodes of Hypoglycemia-Associated ED Visits or Hospitalizations eTable 3. Specialty of Prescribers of Sulfonylurea and/or Insulin Therapy eTable 4. Baseline Regimen by Prescriber Specialty eTable 5. Deintensification of Baseline Regimen by Prescriber Specialty eTable 6. Sensitivity Analysis of Outpatient Cataract Surgery Cohort eFigure. Flow Diagram of Study Population [file jamanetwopen-e2132215-s001.pdf]

Supplementary Online Content

Alexopoulos AS, Kahkoska AR, Pate V, et al. Deintensification of treatment with sulfonylurea and insulin after severe hypoglycemia among older adults with diabetes. *JAMA Netw Open*. 2021;4(11):e2132215. doi:10.1001/jamanetworkopen.2021.32215

eTable 1. *ICD-9* and *ICD-10* Codes Used for Defining Hypoglycemia-Associated ED Visits or Hospitalizations

eTable 2. Characteristics of Individuals With 1 vs Multiple Episodes of Hypoglycemia-Associated ED Visits or Hospitalizations

eTable 3. Specialty of Prescribers of Sulfonylurea and/or Insulin Therapy

eTable 4. Baseline Regimen by Prescriber Specialty

eTable 5. Deintensification of Baseline Regimen by Prescriber Specialty

eTable 6. Sensitivity Analysis of Outpatient Cataract Surgery Cohort

eFigure. Flow Diagram of Study Population

This supplementary material has been provided by the authors to give readers additional information about their work.

© 2021 Alexopoulos AS et al. *JAMA Network Open*.

eTable 1. *ICD-9* and *ICD-10* Codes Used for Defining Hypoglycemia-Associated ED Visits or Hospitalizations

	ICD-9 codes	ICD-10 codes
**Hypoglycemia**	251.0, 251.1, 251.2,	E08.641, E08.649, E09.641,
	270.3, 962.3 or	E09.649, E10.641, E10.649,
	250.8x (and not 259.8,	E11.641, E11.649, E13.641,
	272.7, 681.xx,	E13.649, E15, E16.0, E16.1,
	682.xx, 686.9x, 707.1-	E16.2, T38.3X1A, T38.3X1D,
	707.9, 709.3,	T38.3X1S, T38.3X2A,
	730.0-730.2, 731.8)	T38.3X2D, T38.3X2S,
		T38.3X3A, T38.3X3D,
		T38.3X3S, T38.3X4A, T383X4D,
		T38.3X4S, T38.3X5A, T383X5D,
		T38.3X5S

© 2021 Alexopoulos AS et al. *JAMA Network Open*.

eTable 2. Characteristics of Individuals With 1 vs Multiple Episodes of Hypoglycemia-Associated ED Visits or Hospitalizations

**Characteristic**	**One Event N=58,579**	**Multiple Events N=17,702**
Age, mean (SD)	76.8 (7.73)	75.7 (7.46)
Sex, Male	21,520 (36.7%)	5,821 (32.9%)
**Race**		
White	41,054 (70.1%)	10,869 (61.4%)
Black	11,423 (19.5%)	5,118 (28.9%)
Hispanic	2,650 (4.5%)	805 (4.5%)
Other	3,452 (5.9%)	910 (5.1%)
**Diabetes medications**		
Metformin	20,224 (34.5%)	5,414 (30.6%)
TZD	6,801 (11.6%)	2,396 (13.5%)
DPP-4i	5,294 (9.0%)	1,272 (7.2%)
GLP-1RA	854 (1.5%)	190 (1.1%)
SGLT2 inhibitor	232 (0.4%)	37 (0.2%)
**Baseline Regimen**		
SU Only	22,222 (37.9%)	4,544 (25.7%)
Insulin Only	28,658 (48.9%)	10,587 (59.8%)
SU and Insulin	7,699 (13.1%)	2,571 (14.5%)
Deintensification (outcome)	21,503 (36.7%)	4,504 (25.4%)

Abbreviations: TZD = thiazolidinedione; DPP-4i=dipeptidyl peptidase-4 inhibitor; GLP-1RA=glucagon-like peptide-1 receptor agonist; SGLT2i=sodium/glucose cotransporter-2; SU=sulfonylurea.

© 2021 Alexopoulos AS et al. *JAMA Network Open*.

eTable 3. Specialty of Prescribers of Sulfonylurea and/or Insulin Therapy

	Frequency	Percent
Endocrinologist	10,702	10.07
Other Specialty	12,666	11.91
PCP Only	82,936	78.02

Definitions: Endocrinologist = any baseline fill from an Endocrinologist prescriber (even if there was also a fill from another prescriber type); Other specialist = no baseline fill from an Endocrinologist or Primary Care Physician; PCP only = all baseline fills from Primary Care Physician (PCP).

© 2021 Alexopoulos AS et al. *JAMA Network Open*.

eTable 4. Baseline Regimen by Prescriber Specialty

Baseline Regimen	Endocrinologist N=10,702	Other Specialty N=12,666	PCP Only N=82,936
SU Only	915 (8.5%)	4,107 (32.4%)	27,057 (32.6%)
Insulin Only	8,487 (79.3%)	6,600 (52.1%)	45,267 (54.6%)
SU and Insulin	1,300 (12.1%)	1,959 (15.5%)	10,612 (12.8%)

Abbreviations: SU=sulfonylurea; PCP=Primary Care Physician

© 2021 Alexopoulos AS et al. *JAMA Network Open*.

eTable 5. Deintensification of Baseline Regimen by Prescriber Specialty

Prescriber	Sulfonylurea Use Only	Insulin Use Only	Sulfonylurea and Insulin Use
Endocrinologist	399 (43.6)	2,159 (25.4)	679 (52.2)
Other Specialty	1,803 (43.9)	1,863 (28.2)	1,034 (52.8)
PCP Only	11,993 (44.3)	10,475 (23.1)	4,964 (46.8)

Abbreviations: PCP=Primary Care Physician

© 2021 Alexopoulos AS et al. *JAMA Network Open*.

eTable 6. Sensitivity Analysis of Outpatient Cataract Surgery Cohort

Characteristic		SU Only			Insulin Only			SU+Insulin		
Characteristic	value	N	% Deinten	OR (95% CI)	N	% Deinten	OR (95% CI)	N	% Deinten	OR (95% CI)
Overall		126,294	13.1		89,277	17.5		24,330	28.2	
Event Number	First	81,045	13.6	1.13 (1.09-1.17)	57,134	18.2	1.14 (1.10-1.19)	15,115	28.4	1.03 (0.98-1.10)
	Subsequent	45,249	12.2		32,143	16.3		9,215	27.8	
Age	65-75	71,694	13.7	1.12 (1.09-1.16)	61,271	17.9	1.09 (1.05-1.13)	16,310	28.6	1.06 (0.99-1.12)
	>75	54,600	12.4		28,006	16.7		8,020	27.5	
Sex	Female	71,550	12.9	0.96 (0.93-0.99)	52,973	17.1	0.93 (0.90-0.96)	13,676	27.9	0.97 (0.92-1.03)
	Male	54,744	13.4		36,304	18.2		10,654	28.5	
Race	Asian	4,850	14.2	1.12 (1.03-1.21)	1,665	16.2	0.91 (0.80-1.04)	599	29.4	1.08 (0.90-1.29)
	Black	12,639	13.7	1.07 (1.01-1.13)	12,122	17.7	1.01 (0.96-1.06)	2,674	29.5	1.09 (1.00-1.19)
	Hispanic	4,360	15.3	1.22 (1.13-1.33)	3,358	16.7	0.94 (0.86-1.03)	997	31.0	1.17 (1.02-1.34)
	NAmerN	715	13.1	1.02 (0.82-1.27)	808	15.5	0.86 (0.71-1.04)	208	27.9	1.00 (0.74-1.36)

© 2021 Alexopoulos AS et al. *JAMA Network Open*.

	a t i v e									
	Other	2,758	14.5	1.14 (1.03-1.27)	1,655	19.5	1.13 (1.00-1.28)	447	31.5	1.20 (0.98-1.47)
	Unknown	578	11.8	0.90 (0.70-1.16)	444	15.5	0.86 (0.67-1.12)	130	22.3	0.75 (0.49-1.13)
	White	100,394	12.9		69,225	17.6		19,275	27.8	

© 2021 Alexopoulos AS et al. *JAMA Network Open*.

eFigure 1. Flow Diagram of Study Population

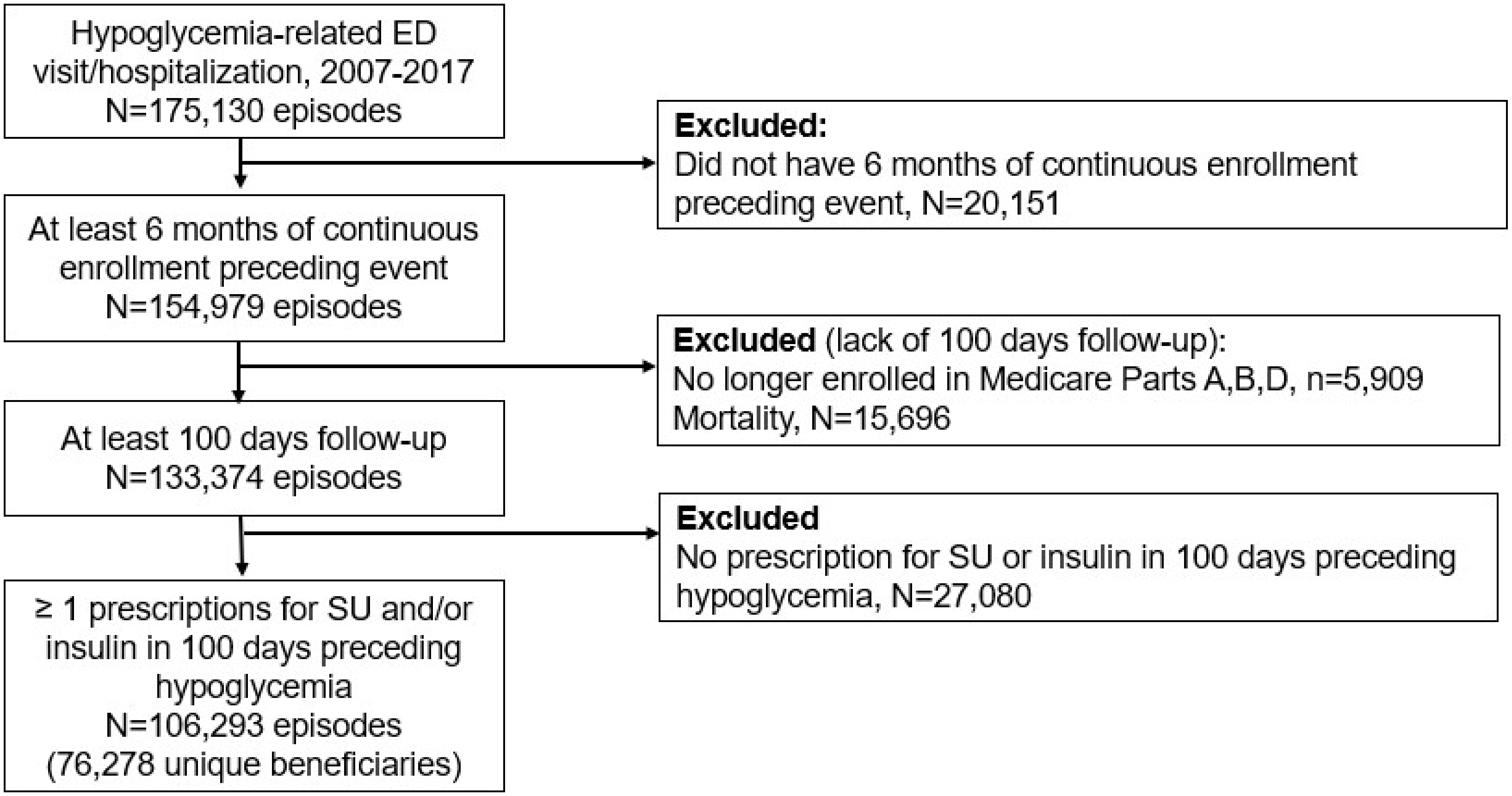

© 2021 Alexopoulos AS et al. *JAMA Network Open*.
